# Supplementary material for: Dynamics of the Apple Fruit Microbiome after Harvest and Implications for Fruit Quality
Source: Microorganisms. 2021 Jan 28;9(2):272. doi: 10.3390/microorganisms9020272 (PMC7912366; doi:10.3390/microorganisms9020272)
Supplement: Supplementary file 1 [file microorganisms-09-00272-s001.zip › Supplementary_figures.docx]

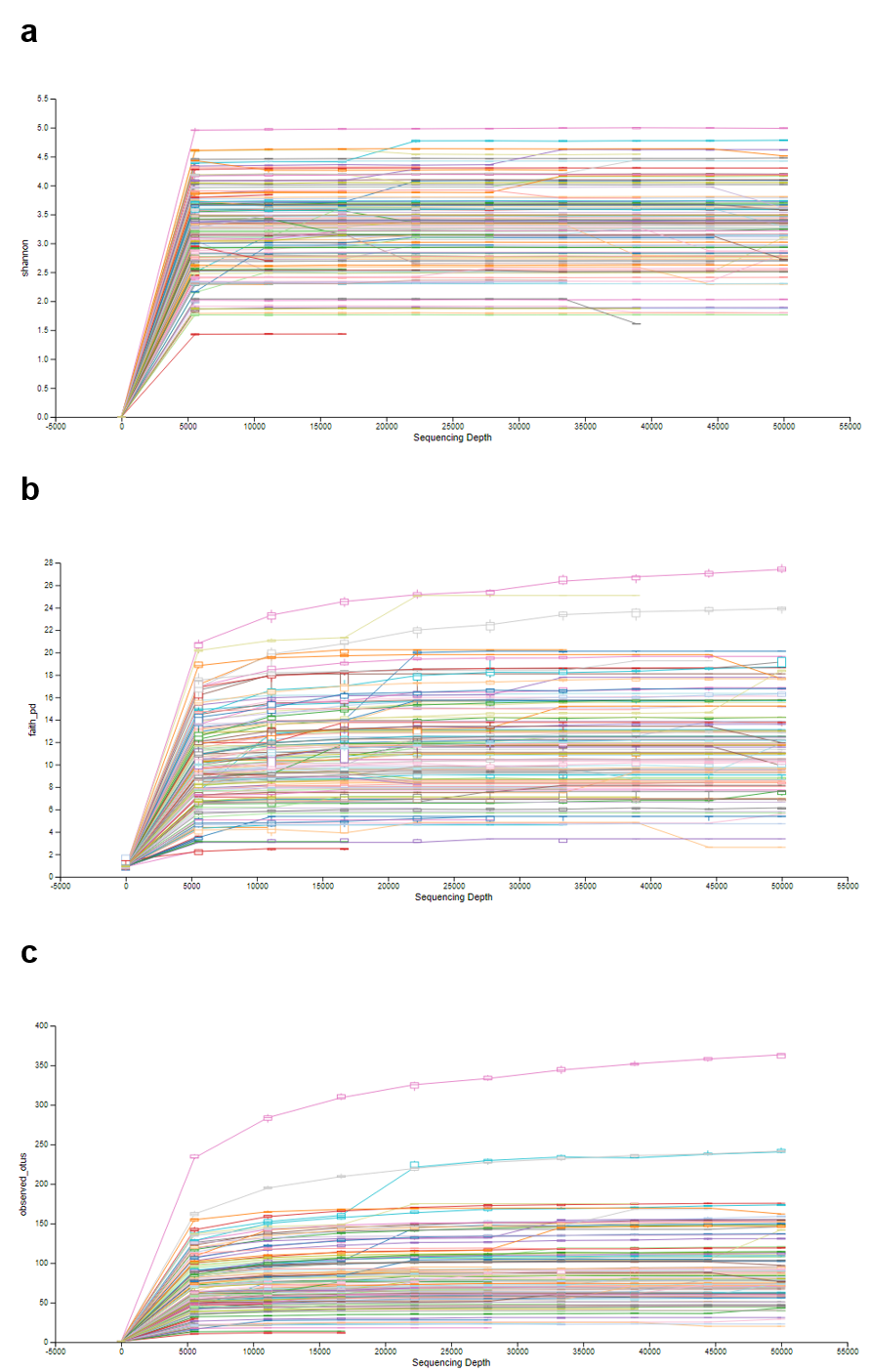


**Figure S1.** Alpha rarefaction plots for all samples and the fungal dataset using parameters Shannon **(a)**, Faith’s PD **(b)** and number of observed OTUs **(c)**.

**
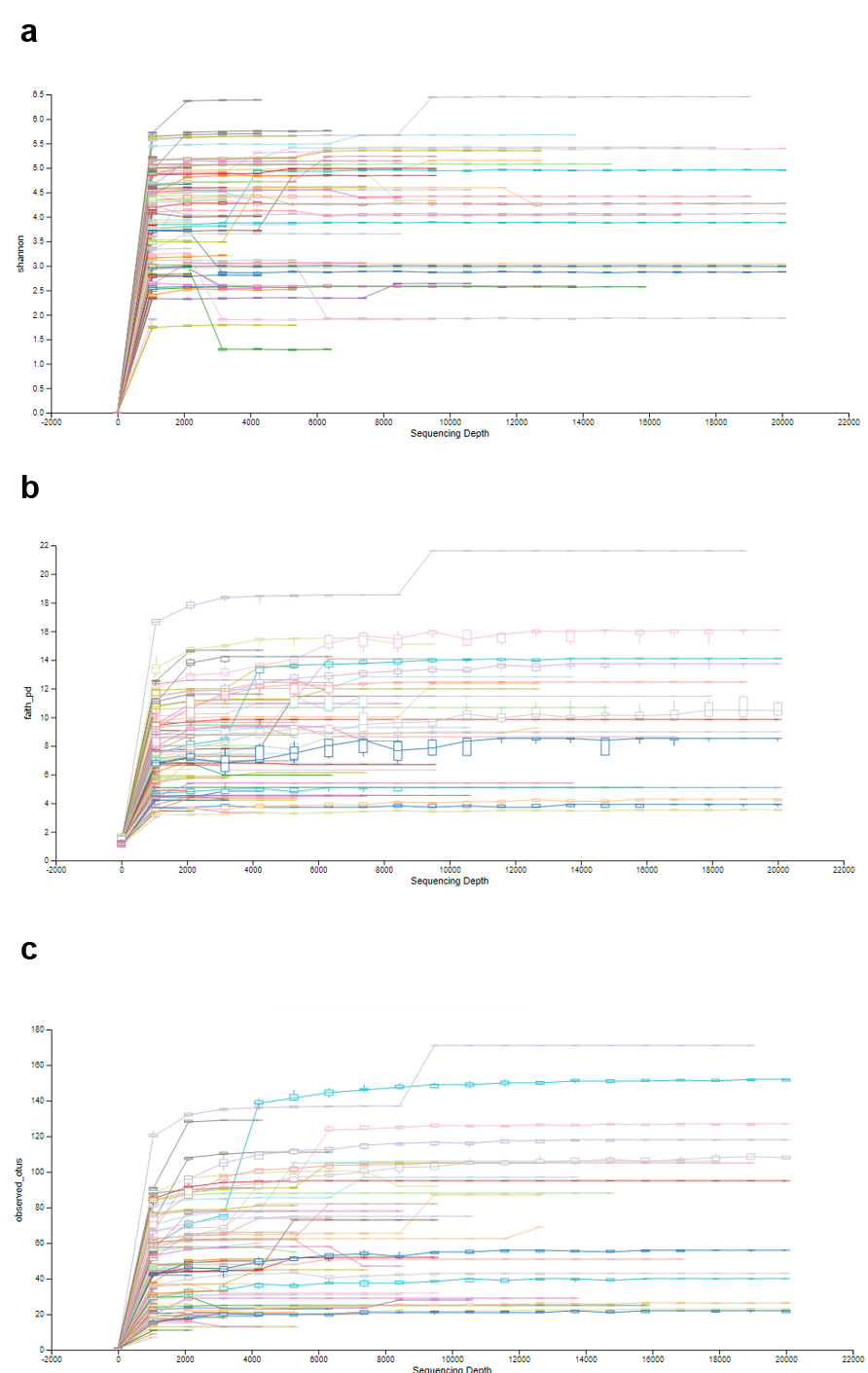
**

**Figure S2.** Alpha rarefaction plots for all samples and the bacterial dataset using parameters Shannon **(a)**, Faith’s PD **(b)** and number of observed OTUs **(c)**.


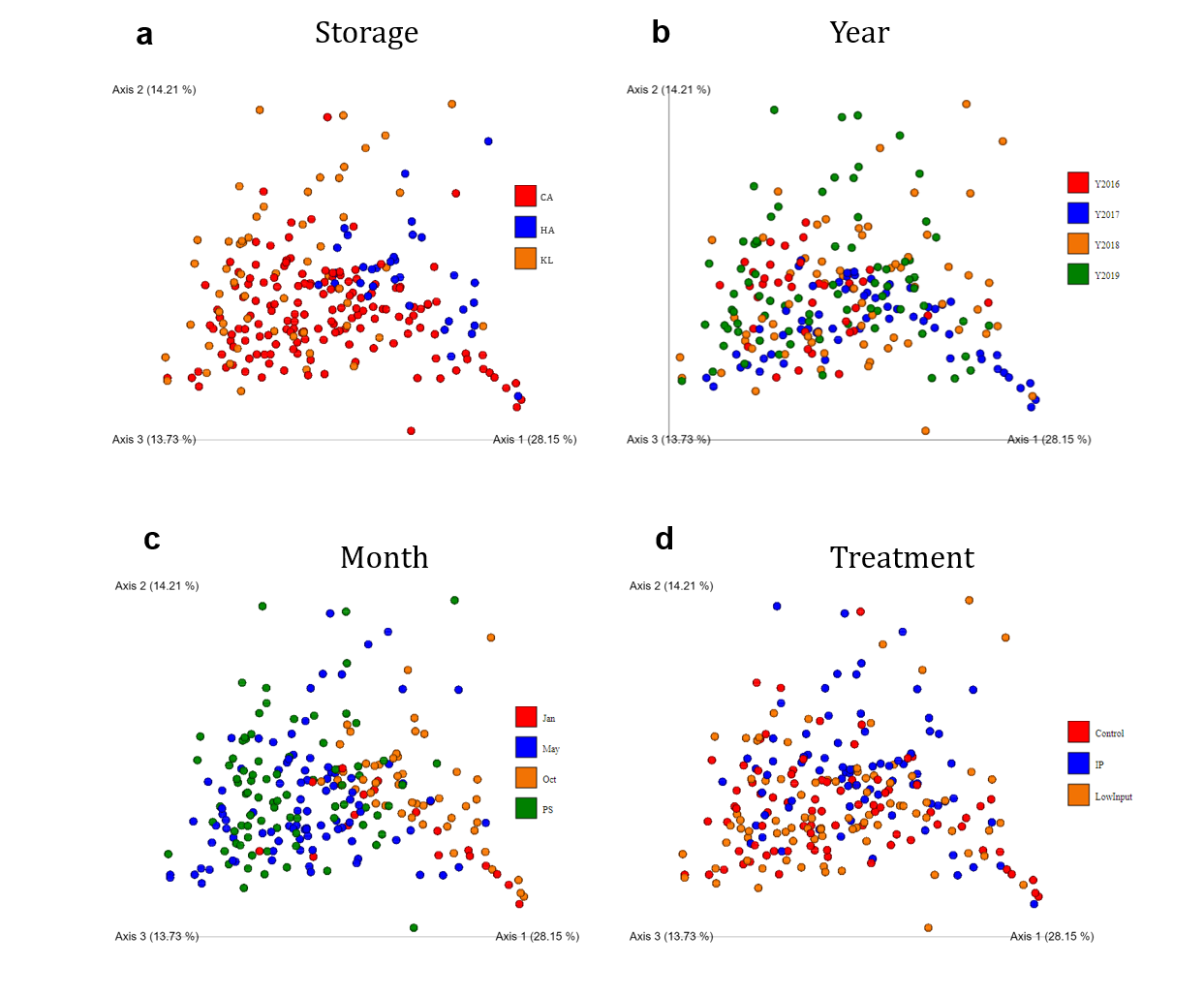


**Figure S3.** Principal Coordinate analysis (PCoA) using weighted UniFrac distance metrics for the fungal subset showing the first two axes labelled by (**a**) Storage condition; (**b**) growing year; (**c**) storage duration; (**d**) plant protection treatment. No clear patterns of clustering were evident in this analysis


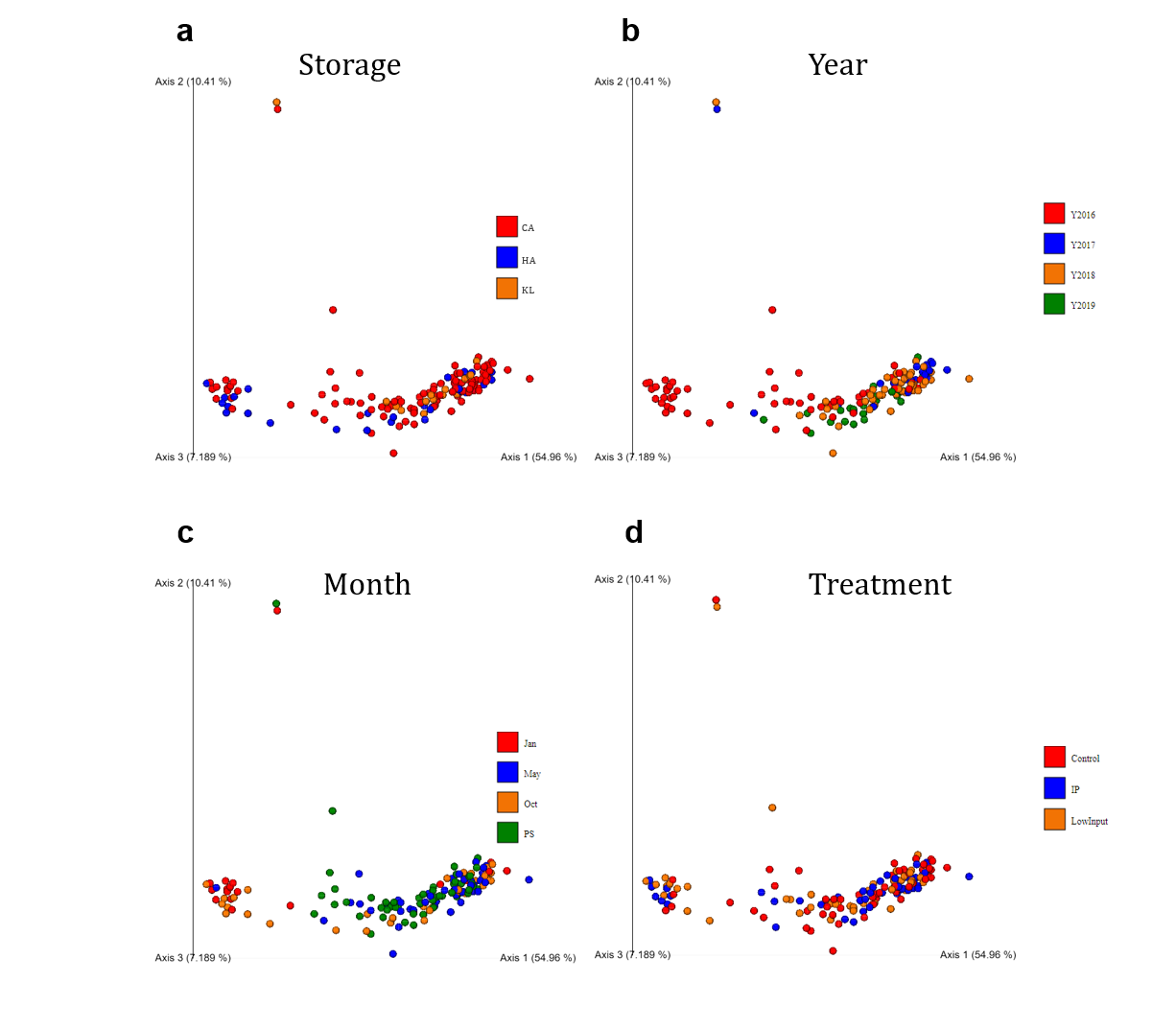


**Figure S4.** Principal Coordinate analysis (PCoA) using weighted UniFrac distance metrics for the bacterial subset showing the first two axes labelled by (**a**) Storage condition; (**b**) growing year; (**c**) storage duration; (**d**) plant protection treatment. No clear patterns of clustering were evident in this analysis

*
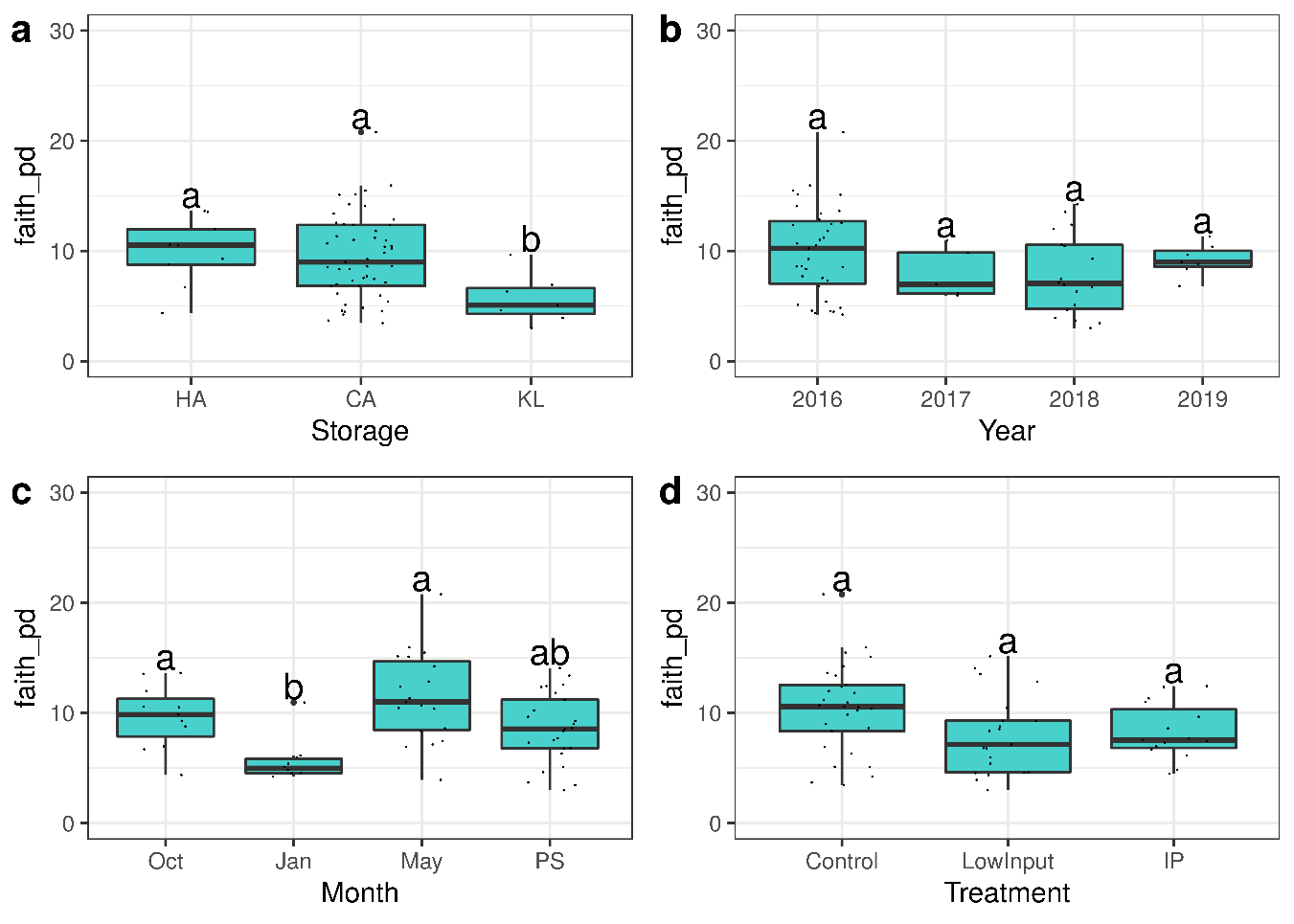
*

**Figure S5.** Alpha diversity measured as faith_pd values of the bacterial microbiome: (**a**) Storage condition; (**b**) growing year; (**c**) storage duration; (**d**) plant protection treatment. No significant effects were measured. This could be due to the low number of reads retained in the analysis.

**
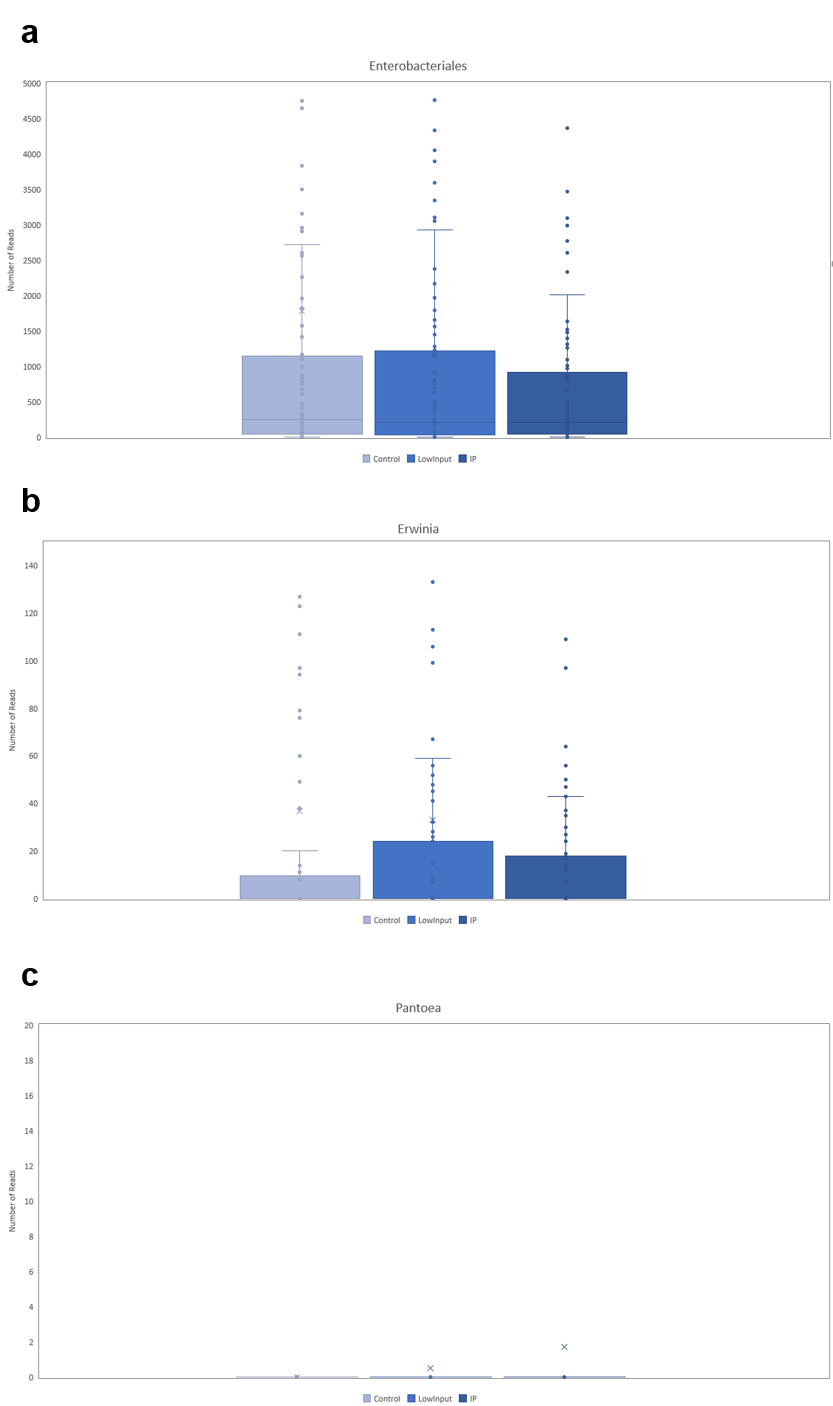
**

**Figure S6.** Abundance of reads assigned to Enterobacteriales **(a)** *Erwinia* **(b)** and *Pantoea* (c) in relation to the plant protection treatments
